# Factors Affecting Unmet Need of Family Planning Among Married Tharu Women of Dang District, Nepal

**DOI:** 10.1155/2018/9312687

**Published:** 2018-09-23

**Authors:** Chet Kant Bhusal, Sigma Bhattarai

**Affiliations:** ^1^Department of Community Medicine, Universal College of Medical Science and Teaching Hospital, Tribhuvan University, Bhairahawa, Rupandehi, Nepal; ^2^Nursing Department, Universal College of Medical Science and Teaching Hospital, Tribhuvan University, Bhairahawa, Rupandehi, Nepal

## Abstract

**Background:**

Increase in population plays a decisive role in providing universal access to reproductive health; however, there is very limited evidence about the reason of unmet need among marginalized and tribal communities such as Tharus. This study aimed to determine the factors affecting unmet need of family planning among married Tharu women of Dang, Nepal.

**Methods:**

Community-based cross-sectional household survey among 650 married Tharu women of age group 15-49 in October 2015 to April 2016 was conducted in Dang district, Nepal. Randomly 3 wards were selected from each Tulsipur municipality, Hekuli Village Development Committee, and Pawan Nagar VDC.

**Results:**

The mean age and parity were 30±7.31 and 2±0.69, respectively. Out of 650 women, 47% were using contraceptives. Westoff model was used for calculating total unmet need which is 49%, where unmet need for limiting and spacing was 27% and 22%, respectively. Hence after combining the current users and total unmet need, total demand for family planning was 96%. After adjustment, significant relation was observed between number of living sons ≥ 1 and unmet need of family planning (OR= 0.4; CI=0.2-0.8,* p*=0.01 ), similarly for women education; lower secondary level (OR=0.3; CI=0.1-0.6,* p*=0.01) and secondary (OR=0.1; CI=0.03-0.4,* p*=0.01); husband education, lower secondary level (OR=0.3; CI=0.1-0.6,* p*=0.01) and secondary (OR=0.4; CI= 0.2-0.9,* p*=0.04); and husband occupation, wage labor (OR=0.6; CI=0.4-0.99,* p*=0.05). In addition, having very good knowledge about method was also significantly associated (OR=0.49; CI= 0.2-0.97,* p*= 0.04).

**Conclusion:**

Unmet need of family planning was significantly higher among less than secondary educated women. It is also predisposed by spouse education, partner's occupation, and number of living sons. This study concerns the need for all stake holders to focus on strategic behavior communication program regarding reproductive health.

## 1. Introduction

For providing universal access to reproductive health, including basic health services, such as antenatal care (ANC), skilled healthcare at childbirth, and information about contraceptive methods, its counseling and supplies increase in population plays a crucial role [1]. Family planning program has been introduced as a key component to deal with rapid population growth and low economic status. In developing countries, an anticipated 225 million women would like to delay or stop bearing child but are not using any method of contraception [2]. Nepal Demographic and Health Survey 2011 revealed that 27 percent of currently married women had not yet met their need [3]. The unmet need of family planning is still high in Nepal, beyond the huge achievements in this sector [4]. Unmet need of family planning is defined as a gap between one's indicated fertility preferences and his or her contraceptives use at a given period [5]. Unmet need is not only limited to whether women/husbands were not provided with family planning facilities; it also means that provided services have not been introduced as an motivating source to women due to lack of adequate information and qualitative services [6]. Males are also recognized to be accountable for the large section of ill reproductive health suffered by their female partners [7, 8]. Men involvement helps not only in accepting a contraceptive but also in its effective use and continuation [8]. Studies have shown that partners who communicate regarding the number of children they want or the use of family planning are more likely to use a contraceptive than those who do not [9, 10]. Community and opinion leaders need to engage in family planning so that they can advocate for the use of FP methods in their community [11]. Wide gap is seen between total demand of family planning and current users. Ultimate goal of family planning program is to reduce the unmet need of family planning, and for this exploring the determinant factors associated with unmet need is required [12].

Tharus constitute 6.75% of total population of Nepal and are indigenous group of inner Terai valleys of Nepal [13].* Kamaiya *is a traditional system of bonded labor common in the western plains of Nepal affecting mostly the indigenous Tharu community [14]. Tharus without land or work could get loans from landowners and were forced to perform slave labor for years and even generations to pay loan for which they had to work on the landowners land as quasi slaves and became marginalized [15]. Marginalized communities are socially excluded groups of people for different reasons, such as age, physical or mental disabilities, economic status, access to education, or living in isolated places or depressed areas [16]. Being marginalized and disadvantaged group, they are economically, socially, educationally, and politically backward and also deprived of various facilities including the health services [17]. Very limited research has been conducted to investigate the reason for unmet need among the marginalized and tribal communities including Tharu in Nepal. Thus this study aims to provide fundamental data to stakeholders and program managers necessary for intervention which will ultimately improve the family planning program in marginalized communities.

## 2. Materials and Methods

### 2.1. Study Design and Source of Population

Community-based cross-sectional study was conducted in Dang district, Nepal, among married Tharu women of age group 15-49 between October 2015 and April 2016. All married Tharu women of reproductive age group 15-49 were included in the study while unmarried or in union Tharu women of reproductive age and women with mental problem were excluded from the study. However women who are not using contraception due to absence of husband for more than one year were included during data collection but not included in analysis of unmet need of contraception.

### 2.2. Sample Size Determination and Sampling Technique

Sample size was 650 married women, which was determined by using formula N= Z^2^pq/L^2^ [18] with 95% level of confidence interval, 3.5% margin of error, 5% nonresponse rate, and 27% of women estimated to have unmet need [3]. Tulsipur municipality out of 4 municipalities and Hekuli and Pawan Nagar VDCs out of 31 VDCs were selected purposively as they cover large portion of Tharu population. Further 3 out of 20 wards from Tulsipur municipality and 3 out of 9 wards from both VDCs, i.e., Hekuli and Pawan Nagar, were selected randomly for data collection. Then house to house survey was used to trace out respondents in communities.

### 2.3. Data Collection Procedures and Validity

Face to face interview was performed using pretested semistructured interview schedule for collecting data. The questionnaire was translated into Nepali, then into English, and again into Tharu language to find misinterpretation, and then correction was made. Five data collectors, including one principal investigator with qualification of Master's in public health as well as Master's in sociology and four enumerators with qualification of Bachelor's in public health/nursing, were involved in data collection.

### 2.4. Data Processing and Analysis

Data checking, compiling, and editing were performed manually by the researcher. Collected data were coded, entered into Microsoft Excel, and cleaned, and then further analysis was done by SPSS version 17 software package. Simple descriptive statistics such as frequencies, means, and standard deviations were calculated, and associated factors between the different variables in relation to the outcome variable were measured by chi-square test having odds ratio with 95% confidence interval. Bivariate analysis was used primarily to check whether variables have association with the dependent variable individually, and multivariate logistic regression (stepwise backward likelihood ratio method) was conducted to analyze factors which were associated with unmet need of family planning, while assessing for multicollinearity. All variables found to be associated with the main outcome variables by having odds ratio reaching statistical significance in the bivariate model (*p*<0.05) were nominees for the multivariable model at 95% CI (*p*<0.05). The data were summarized, adjusted odds ratios (AORs) were estimated, and their corresponding value at 95% confidence intervals (95% CI) was computed.

### 2.5. Setting

Dang district is located in the Inner Terai and Middle Hills of Rapti zone in the Mid-Western Development Region of Nepal. The district is adjacent to Salyan and Rolpa in the north; India in the south; Kapilvastu, Arghakhanchi, and Pyuthan in the east; and Surkhet and Banka districts in the west. At the time of study, there were 4 municipalities, 5 electoral consistencies, and 31 VDCs, among which 5 VDCs lie in hilly region [19]. According to Central Bureau of Statistics 2011 total population of Dang district was 552,583, among which 291,524 (52.76%) were female and 261,051 (47.24%) were male. Annual population growth rate of Dang district was 1.78 [20]. Among total population, 29.52% were Tharus, where 30.27% and 28.85% were male and female Tharus, respectively [19].

### 2.6. Concept of Unmet Need of Family Planning

Unmet need of married women was introduced in this study, which was estimated using Westoff model [21]. Total demand for family planning was calculated by sum of the percent of unmet need and the percent of using contraception. The surveyed women were first divided into two groups, i.e., those who had used contraceptives and those who had not used those methods. The nonusers were then subdivided into pregnant or amenorrheic women and nonpregnant or nonamenorrheic categorized at the time of the survey. The pregnant or amenorrheic women were further subdivided into three categories, i.e., those whose pregnancy was intended, mistimed, and unwanted at the time of survey. Those who had mistimed and unwanted pregnancy were regarded as one component of the total unmet need. The other component consists of those who are neither pregnant nor amenorrheic. Further they were divided into fecund or infecund; fecund women were again subdivided by their reproductive preference into 3 categories, i.e., those who want pregnancy soon (not included in unmet need), want no more pregnancy, and want pregnancy later.

## 3. Results and Discussion

### 3.1. Results

The mean age and parity were 30 ±7.31 and 2 ± 0.69, respectively. About 43% of women and 19% of their husbands were below 18 years during their marriage. Almost one-fifth (21%) of respondents were found still unable to read or write and slightly more, i.e., 23%, had their informal class known as* Praoudh Sickshya* (Table 1).

Most of the respondents, i.e., 98%, had heard about family planning methods. Regarding the users of family planning, slightly less than two-fifth (39%) of the women and 8% of their husbands were using family planning during the survey. Among 255 respondents who had used family planning, nearly one-third (30%) had used the permanent method, and among 50 husbands more than half (54%) had used condom. More than two-thirds of the women and their husbands (68%) obtain family planning devices from government health facilities (Table 2).

Among 62 pregnant women, 16% want to do abortion. Major reason for not using of family planning method was due to fear of side effects (42%), and inaccessibility was 1% (Table 3).

**Figure 1:**
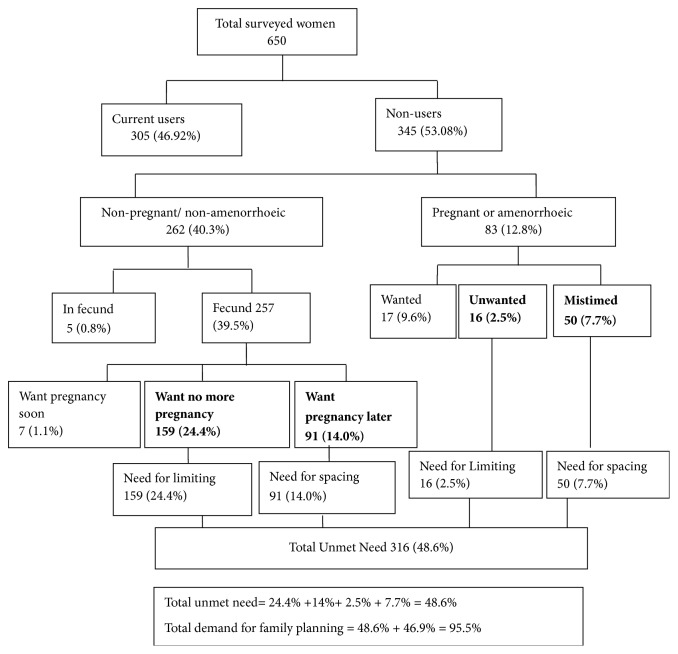
**Unmet need for contraception (Westoff model)**.** Note.** Fifteen respondents who are not using any contraceptive methods with the reasons of absence of husbands from last one year are excluded from the total unmet need.

Figure 1 illustrates the unmet need for contraception (Westoff model). During the survey 47% (305/650) of women were using contraceptive. Unmet need for limiting was 27% while unmet need for spacing was 22%. By using Westoff model the total unmet need was estimated as 49%. Likewise total demand for family planning was 96% (Figure 1).

Table 4 represents logistic regression model where number of living sons ≥ 1 and its relation with unmet need of family planning (OR= 0.4; CI=0.2-0.8,* p*=0.01), similarly for women education, lower secondary level (OR=0.3; CI=0.1-0.6,* p*=0.01) and secondary (OR=0.1; CI=0.03-0.4,* p*=0.01); husband education, lower secondary level (OR=0.3; CI=0.1-0.6,* p*=0.01) and secondary (OR=0.4; CI= 0.2-0.9,* p*=0.04); and husband occupation, wage labor (OR=0.6; CI=0.4-0.99,* p*=0.05). Significant associated factors with knowledge about method of family planning were found: having very good knowledge was 0.49 (OR=0.49; CI=0.2-0.97,* p*=0.04). However, some variables such as religion, women's age at marriage, place of residence, birth interval of child, fear of side effects, husbands'/family members' objection, inconvenience to use, distance to reach family planning, and time to reach family planning were not found significant with unmet need. After subjected to multivariate model, age, family size, family type, number of living children, sex of youngest child, women occupation, earning status of women, and knowledge about sources of family planning were not associated with total unmet need for family planning, which were seen significant in bivariate analysis (Table 4).

### 3.2. Discussion

The finding of this study revealed that 49% of respondents had unmet need of family planning which was higher than the national (27%) and regional unmet need (10.9 %) in 2011 and 2014, respectively [3, 22]. Likewise, the study conducted in a district of eastern Nepal documented a lower (25%) unmet need [4] than the present study. The difference might be due to Tharu being marginalized community. However several studies conducted in tribal and rural communities, such as study of Das S et al. in West Bengal of India, Chouhary S et al. in rural area of Haryana, Bhattacharya SK et al. in Kolkata India, Girma Temam Shifa and Mekdes Kondale in Southern Ethopia, and Ali AA and Okud A in eastern Sudan, found similar result [23–27]. Small proportion was observed for unmet need for spacing in comparison with that for limiting (22 versus 27). This interpretation found similarity with survey conducted by Nepal Demographic and Health Survey 2011 and 2016 and other studies conducted in low-income countries such as Nepal and India where unmet need for limiting contributed high proportion [3, 4, 24, 25, 28].

The present study revealed that 47% of the couples of respondents were using a family planning method that is inconsistent with Nepal Demographic and Health Survey report which shows higher (53%) contraceptive rate including modern as well as natural method [28]. However this is relatively high contraceptive rate in this type of tribal society. This may be due to expansion of equitable access and utilization of high-quality family planning services, strengthening public and private sector health systems, and increasing the availability of modern family planning methods by Government of Nepal [29], though similar result was found in a study conducted by Prusty RK in 2014 where 45% of Hindu tribal women in India used modern contraceptives [30]. The total demand for family planning in the current study is 96% which is higher than the national level [3, 28]. This divergence emerged because of the high prevalence of unmet need for family planning among the disadvantaged community.

In the current study statistical association was established between number of living sons and unmet need which is also supported by studies done by Bhandari GP in eastern Nepal, Nazir S et al. in India, and Dahal GP in Nepal [4, 31, 32]. The present study revealed that women and husband education was statistically associated with unmet need. Similar pattern follows in studies conducted in West Bengal of India, Kassala state of Eastern Sudan, Oromia region of Ethiopia, Nigeria, and Bangladesh but the study contradicts with a study by Anthony OI et al. in southeast Nigeria [23, 27, 33–36]. Statistical association was established between husband's occupation and unmet need. Similar result was found in study done at Tamil Nadu, India, but the study contradicts with a study by Anthony OI et al. in southeast Nigeria [36, 37]. The study found significant association of unmet need of family planning with knowledge about method of family planning. This observation is in agreement with other studies from different low-income countries, such as West Bengal of India, Pakistan, and Eritrea Demographic and Health Survey 2002 [23, 38, 39].

Major reason for not using family planning in this study was found as fear of side effects (42%). This is in accordance with a study conducted by Mustafa G et al. in rural areas of Pakistan and Govindasamy and Boadiin in Ghana [40, 41]. About one-fourth (26%) of the women in the current study were not using family planning due to the husband objection and 14% due to objection of family members, in concordance with the study conducted by Bhattacharya SK et al. in Kolkata, India, where main reason for unmet need was opposition from husband, family, and community (32%) [25]; however few percentages were found in study done by Ebrahim SM and Muhammed NK in Basrah city in south of Iraq [42].

## 4. Conclusions

Majority of women had heard about family planning methods. However, few had practiced them, which results in high unmet need among married Tharu women of Dang district. The gap between knowledge and practice regarding family planning needs serious attention from the concerned authority to be addressed. The contraceptive prevalence rate of Dang district will be doubled if those women with unmet need also used any method of family planning. In this context, this study concerns the need for the policy maker, government officials, and program managers to focus on strategic behavior communication program regarding reproductive health including family planning among tribal communities like Tharus.

## Figures and Tables

**Table 1:** Distribution of background related characteristics of study population.

**General Characteristics**	**Frequency (n=650)**	**Percentage**
**Age of women at marriage**		
Below 18	281	43.2
18 and above	369	56.8
Mean age ± SD	17.94±2.46	
**Age of husband at marriage**		
Below 18	125	19.2
18 and above	525	80.8
Mean age ± SD	19.75±2.90	
**Educational Status of Women**		
Illiterate	133	20.5
Literate	149	22.9
Primary	116	17.8
Lower Secondary	164	25.2
Secondary	48	7.4
SLC and above	40	6.2
**Husband's Educational Status**		
Illiterate	95	14.6
Literate	57	8.8
Primary	135	20.8
Lower Secondary	200	30.8
Secondary	88	13.5
SLC and Above	75	11.5

**Table 2:** Distribution of family planning method-related characteristics of study population.

**Characteristics**	**Frequency (n=650)**	**Percentage**
**Family Planning user (women)**	255	39.2
**Method used (n=255)**		
Oral Pills	25	9.8
IUCD	38	14.9
Depo-Provera	72	28.2
Implant/Norplant	33	13.0
Female Condom	2	0.8
Sterilization (Permanent)	77	30.2
Natural Method	8	3.1
**Family Planning user (husbands)**	50	7.7
**Method used (n=50)**		
Condom	27	54.0
Vasectomy (Permanent)	19	38.0
Male Withdrawal	4	8.0
**Sources of obtaining family planning devices** **∗**		
Government Health facilities	443	68.2
FCHVs	133	20.5
Medical shops	207	31.8
Meri-stopes	30	4.6
Traditional Healers	6	0.9

**∗** indicates multiple responses given by respondents.

**Table 3:** Distribution of common reasons for not using family planning methods of study population.

**Reason for not using family planning**	**Number (n=329)**	**Percentage**
Inaccessibility	4	1.2
Fear of side effect	137	41.6
Objection of husband	85	25.8
Objection of family members	46	14.0
Son preference	52	15.8
Lack of information about method	58	17.6
Lack of counseling from health worker	37	11.3
Due to rumors about family planning	23	7.0
Inconvenience to use	37	11.3
Lack of time	21	6.4
Small baby	8	2.4
Husband not in home	15	4.5

Note. Multiple responses given by respondents.

**Table 4:** Factors affecting unmet need for family planning in using bivariate and multivariate analysis.

**Characteristics**	**Unadjusted OR** **(95**%** CI)**	**p-value**	**Adjusted OR** **(95**%** CI)**	**p-value**
**Age of women**				
≤ 18 years	1		1	
19-29 years	0.38(0.13-1.06)	0.003	2.83(0.38-21.39)	0.313
30-40 years	0.24(0.09-0.68)		1.01(0.12-8.20)	0.993
≥ 41 years	0.23(0.07-0.70)		0.36(0.04-3.29)	0.366
**Family Size**				
≤ 5 members	1		1	
> 5 members	1.45(1.05-1.99)	0.023	0.78(0.35-1.48)	0.368
**Family Type**				
Single	1		1	
Joint and extended	1.66(1.21-2.28)	0.002	1.47(0.73-3. 2.99)	0.285
**No of live children**				
One	1		1	
Two to three	0.49(0.33-0.72)	<0.001	0.75(0.41-1.37)	0.346
> 3	1.31(0.78-2.18)		1.44(0.60-3.44)	0.417
**No of living sons**				
0	1		1	
≥ 1	0.43(0.28-0.67)	<0.001	**0.41(0.21-0.81)**	**0.010** **∗**
**Sex of youngest child**				
Male	1		1	
Female	1.39(1.01-1.92)	0.045	0.88(0.55-1.41)	0.593
**Women's education**				
Illiterate	1		1	
Literate	0.78 (0.48-1.26)	<0.001	0.79(0.45-1.43)	0.445
Primary	1.28 (0.75-2.17)		1.15 (0.56-2.37)	0.699
Lower Secondary	0.25(0.15-.40 )		**0.27 (0.13-0.57)**	**0.001** **∗**
Secondary	0.12(0.05-0.28)		**0.12(0.06-0.40)**	**0.001** **∗**
SLC and above	0.25(0.11-0.55)		0.31 (0.08-1.27)	0.104
**Husband's education**				
Illiterate	1		1	
Literate	0.87(0.43-1.74)	<0.001	0.92(0.42-2.02)	0.830
Primary	0.81(0.47-1.42		0.64(0.33-1.25)	0.189
Lower Secondary	0.32(0.19-0.53)		**0.30(0.14-0.61)**	**0.001** **∗**
Secondary	0.24(0.13-0.45)		**0.39( 0.17-0.94)**	**0.035** **∗**
SLC and above	0.22(0.11-0.43)		0.59 (0.19-1.80)	0.354
**Women's Occupation**				
Agriculture	1		1	
Business and Services	0.20(0.09-0.45)	<0.001	0.19(0.04-1.09)	0.062
Wage Labor	0.34(0.13-0.89)		0.25(0.04-1.44)	0.121
Housewife	1.35(0.95-1.92)		1.10(0.70-1.73)	0.671
**Husband's Occupation**				
Agriculture	1		1	
Business and Service	0.44(0.28-0.69)	<0.001	0.86(0.45-1.65)	0.648
Wage Labor	0.48(0.33-0.70)		**0.61(0.37-0.99)**	**0.049** **∗**
Foreign Employee	0.65(0.31-1.36)		1.55(0.62-3.93)	0.351
**Earning status of Women**				
No	1		1	
Yes	0.32(0.19-0.56)	<0.001	1.49 (0.36-6.20)	0.584
**Knowledge about sources of FP**				
Poor knowledge	**1**		1	
Knowledge	0.76(0.55-1.06)	0.026	1.10(0.72-1.68)	0.675
Very good knowledge	0.39(0.18-0.82)		1.65 (0.62-4.39)	0.320
**Knowledge about method of FP**				
Poor Knowledge	1		1	
Knowledge	0.94(0.55-1.59)	<0.001	1.31 (0.68-2.52)	0.414
Very Good knowledge	0.32(0.19-0.54)		**0.49 (0.25-0.98)**	**.043** **∗**

*∗*Significant (p<0.05) AOR in bold denotes significant.

## Data Availability

The data used to support the findings of this study are available from the corresponding author upon request.

## Abbreviations

ANC: Antenatal care
AOR: Adjusted odds ratio
CI: Confidence interval
CPR: Contraceptive prevalence rate
OR: Odds ratio
SPSS: Statistical package for the social sciences
UNICEF: United Nations Children Fund
VDC: Village Development Committee.

## Additional Points

*Policy Implications*. Even though Government of Nepal had tried to include Social Inclusive Group in different plans and policies, but very few data/information was available among tribal population. The findings of this study assured the need for formulation of strategic behavior communication programs for targeted marginalized group such as Tharus.* Areas of Further Research*. Further studies need to be done in a large scale with the help of qualitative research method to explore cultural practice, belief, and value system related to family planning among disadvantaged and marginalized communities.* Limitations*. The study did not represent unmarried women or women who are in union because of the fact that community reject the sexual activities outside marriage in a developing country like Nepal.
